# Quantitative analysis of PPT1 interactome in human neuroblastoma cells

**DOI:** 10.1016/j.dib.2015.05.016

**Published:** 2015-06-12

**Authors:** Enzo Scifo, Agnieszka Szwajda, Rabah Soliymani, Francesco Pezzini, Marzia Bianchi, Arvydas Dapkunas, Janusz Dębski, Kristiina Uusi-Rauva, Michał Dadlez, Anne-Claude Gingras, Jaana Tyynelä, Alessandro Simonati, Anu Jalanko, Marc H. Baumann, Maciej Lalowski

**Affiliations:** a Meilahti Clinical Proteomics Core Facility, Institute of Biomedicine, Biochemistry and Developmental Biology, University of Helsinki, Helsinki, Finland; b Institute for Molecular Medicine (FIMM), University of Helsinki, Helsinki, Finland; c Department of Neurological and Movement Sciences, University of Verona, Verona, Italy; d Unit for Neuromuscular and Neurodegenerative Disorders, Laboratory of Molecular Medicine, Bambino Gesù Children’s Hospital, IRCCS, Rome, Italy; e Mass Spectrometry Laboratory, Department of Biophysics, Institute of Biochemistry and Biophysics, Polish Academy of Sciences, Warsaw, Poland; f Folkhälsan Institute of Genetics, Helsinki, Finland; g National Institute for Health and Welfare, Public Health Genomics Unit, Helsinki, Finland; h Centre for Systems Biology, Samuel Lunenfeld Research Institute at Mount Sinai Hospital, Toronto, and Department of Molecular Genetics, University of Toronto, Toronto, ON, Canada; i Doctoral Program Brain & Mind, University of Helsinki, Helsinki, Finland

## Abstract

Mutations in the *CLN1* gene that encodes Palmitoyl protein thioesterase 1 (PPT1) or CLN1, cause Infantile NCL (INCL, MIM#256730). PPT1 removes long fatty acid chains such as palmitate from modified cysteine residues of proteins. The data shown here result from isolated protein complexes from PPT1-expressing SH-SY5Y stable cells that were subjected to single step affinity purification coupled to mass spectrometry (AP-MS). Prior to the MS analysis, we utilised a modified filter-aided sample preparation (FASP) protocol. Based on label free quantitative analysis of the data by *SAINT,* 23 PPT1 interacting partners (IP) were identified. A dense connectivity in PPT1 network was further revealed by functional coupling and extended network analyses, linking it to mitochondrial ATP synthesis coupled protein transport and thioester biosynthetic process. Moreover, the terms: inhibition of organismal death, movement disorders and concentration of lipid were predicted to be altered in the PPT1 network. Data presented here are related to Scifo et al. (J. Proteomics, 123 (2015) 42–53).

**Specifications table**

**Table:** 

Subject area	Biology, Proteomics, Neuroscience
More specific subject area	Neuronal Ceroid Lipofuscinoses
Type of data	Excel Tables (MS data analysis, GO analysis) and figures
How data was acquired	*Mass spectrometry* (Q Exactive Hybrid Quadrupole-Orbitrap mass spectrometer, Thermo Scientific)*, SAINT analysis (*version exp3.3*), Confocal microscopy* (Leica SP8 confocal microscope; Wetzlar, Germany), *qPCR (*ABI 7500Fast*) and Western blot analysis*
Data format	Raw, filtered and analyzed
Experimental factors	Brief description of any pretreatment of samples
Experimental features	N/A
Data source location	N/A
Data accessibility	Data is with this article

**Value of the data**

• Novel knowledge of the PPT1 interactome in SH-SY5Y cells
• Label-free quantitation of PPT1 IP to determine their relative abundances
• Identification of novel PPT1 IP and insight into novel roles of PPT1
• Identification of putative PPT1 substrates
• Preliminary dataset for exploring functional relationships in the PPT1 network

## 1 Data, experimental design, materials and methods

In order to determine PPT1 IP, we first cloned full length human PPT1 into CTAP-Puro (a mammalian retroviral based expression vectors) and generated PPT1 expressing SH-SY5Y stable cells by retroviral infection. We then isolated PPT1 complexes from the stable cells by Single-step Affinity purification, subjected them to filter-aided sample preparation (FASP) and performed mass spectrometry analysis on a *Q* Exactive Hybrid Quadrupole-Orbitrap instrument. Based on label free quantitation of the MS data by SAINT platform, we identified 23 PPT1 interacting partners (IP).

### 1.1 Retroviral production, transduction and generation of stable cell lines

**Fig. 1:**
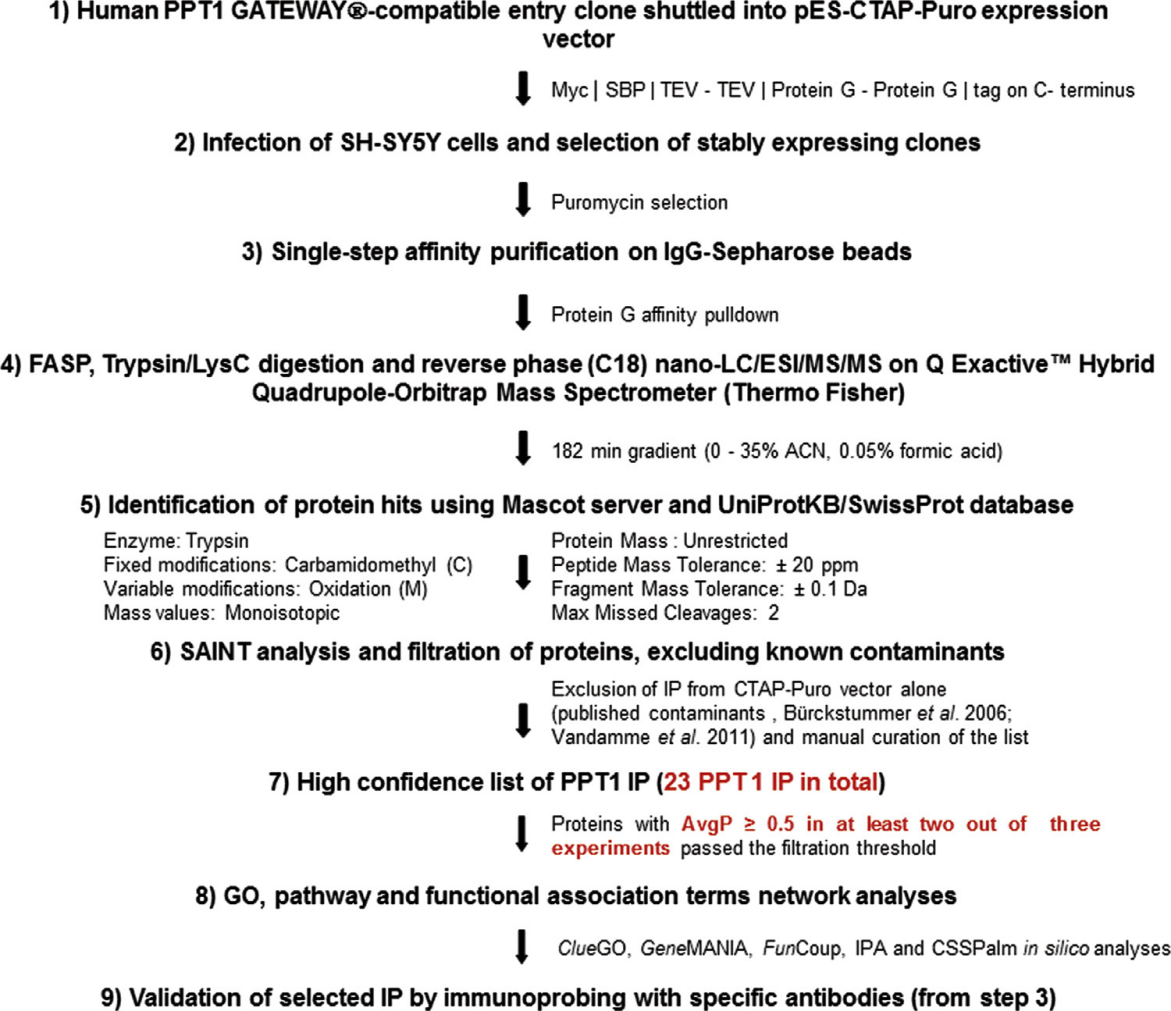
Flow-chart depicting various steps of affinity purification and analysis of PPT1 interacting partners. Human PPT1 Gateway^®^-compatible entry clone was shuttled into pES-CTAP-Puro destination vector [8], to generate mammalian expression vector used to infect SH-SY5Y cells. Stably expressing clones were isolated by Puromycin selection, expanded and subjected to single step Protein G affinity purification. Following FASP and Lys-C/Trypsin digestion the corresponding peptides were separated by nano-LC-ESI tandem mass spectrometry and analysed by bioinformatics tools (Mascot and SAINT, respectively), prior to filtration of known contaminants. High confidence set containing identified PPT1 interacting proteins underwent Gene Ontology, pathway and functional association terms network analysis to select targets for further biochemical and functional validation.

For producing retroviral particles, DNA of retroviral vectors was introduced into HEK 293T cells [2,3] simultaneously with two packaging plasmids: pCMV-Gag-Pol vector (expresses gag protein and reverse transcriptase of Moloney murine leukemia virus) [4] and pVSV-G (expresses protein G of vesicular stomatitis virus) [5]. The three plasmids were mixed in proportions of 7.5:5:1, respectively and introduced into cells using the calcium phosphate method according to the manufacturer’s protocol (Life Technologies Europe BV). Cell-free virus was harvested two days post transfection, filtered through 0.45 μm pore size filter (Millex-HV Filter Unit, Millipore, Ireland Ltd.) and used to infect low passage (P5-10) SH-SY5Y cells. Cells were grown in DMEM: F12 Ham’s media (1:1), supplemented with Penicillin (100 µg/ml), Streptomycin (100 µg/ml), Glutamine, non-essential amino acids (1×) and 10% FBS (Life Technologies Europe BV), at 37 °C under humidified atmosphere of 95% air and 5% CO_2_. Stable integrants were isolated by puromycin selection with 1.5 µg/ml and confirmed using immunocytochemistry and Western blot analysis (see Figs. 1 and 2 in [1]). Stably infected cells were maintained in an undifferentiated state (.80% confluence) and constantly checked for consistent growth rates and morphological features.

### 1.2 Expression of untagged human PPT1 in the stably transfected SH-SY5Y cells

SH SY5Y cells were transfected either with the empty vector pcDNA3 (Invitrogen, LT) or with the vector carrying CLN1 wild-type gene (CLN1 wt). Cells were cultured in DMEM medium supplemented with 600 μg/ml G-418 to select for clones stably expressing the constructs. qPCR experiments using inventoried Taqman assay (Applied Biosystems, Life Technologies) were performed to assess the expression of CLN1 mRNA (Hs00165579_m1); GAPDH (Hs99999905_m1) was used as reference endogenous gene. cDNAs were amplified in a ABI 7500Fast real time PCR. Enzymatic activity was assessed as previously described in materials and methods. For Western Blotting analysis, cells from the 3 different cell lines were collected and lysed in RIPA buffer. 40 μg of proteins were separated by SDS-PAGE and further processed for immunoblotting analysis.

### 1.3 Immunofluorescence microscopy

SH-SY5Y-PPT1-CTAP-Puro stable cells grown on coverslip glasses were fixed after 48 h with ice cold methanol prior to blocking with PBS containing 0.5% BSA (PBS-B). The cells were incubated overnight at +4 °C with primary antibodies: rabbit polyclonal anti-human-PPT1 (1:300) [6], rabbit polyclonal anti-PPT1 (HPA021546, 1:500; Sigma) and mouse monoclonal anti-LAMP 1 (H4A3, 1:100, Developmental Studies Hybridoma Bank, Iowa). They were subsequently washed 3 times with PBS-B and incubated for 1 h at room temperature with secondary antibodies: goat anti-mouse AlexaFluor-488 and goat anti-rabbit AlexaFluor-568 (Life Technologies Europe BV). The coverslips were washed 3 times with PBS-B and counter-stained with Hoechst 33342 (10 µg/ml) for 10 min (Life Technologies Europe BV). The coverslips were finally washed twice with PBS, rinsed with deionized water and mounted with Moviol/glycerol mounting media supplemented with 2.5% (v/w) DABCO. Images were acquired using a Leica SP8 confocal microscope (Wetzlar, Germany) and deconvoluted using Huygens Professional (Scientific Volume imaging, Hilversum). Analysis of co-localisation was performed with coloc2 plugin in FIJI image analysis software [7]. Co-localisation was presented as percentage of Mander’s overlap coefficient.

### 1.4 Western blotting and antibodies

Protein samples were separated by standard electrophoresis protocols using homogeneous gels containing 12% polyacrylamide, 0.1% SDS (SDS-PAGE) under reducing conditions or on gradient Bis-Tris 4–12% NuPAGE® gels in MES SDS running buffer (Novex; Life Technologies, USA) and transferred to nitrocellulose membranes (Perkin-Elmer Finland Oy, Turku, Finland). Membranes were blocked with 3% BSA in TBST buffer (0.01 M Tris–HCl, pH 8.0, 0.15 M NaCl, and 0.1% Tween 20) and incubated with the following primary Abs: rabbit polyclonal anti-human-PPT1 (1:500) [6], rabbit polyclonal anti-PPT1 (HPA021546, 1:1000) (Sigma-Aldrich Finland Oy); mouse monoclonal anti-VCP [#612182] (1:1000) (BD Transduction Laboratories^TM^); mouse monoclonal anti-ATP5B [clone 3D5AB1] (A21351, 1:1000) (Molecular Probes/Life Technologies Europe BV, Espoo, Finland); rabbit polyclonal anti-DBH (NBP1-31386, 1:1500) (Novus Biologicals, Cambridge, UK); mouse monoclonal anti-Myc [9E10] (ab32, 1:1500) and mouse monoclonal anti-β-actin [AC15] (A1978, 1:2000) (Sigma-Aldrich Finland Oy). Each antibody was used according to the manufacturer’s protocols. After extensively washing with TBST, the primary antibodies were detected by the appropriate horseradish peroxidase-conjugated secondary antibodies (Bio-Rad Laboratories AB, Sundbyberg, Sweden) and revealed by chemiluminescence coupled to autoradiography.

### 1.5 Detection of endogenous PPT1 interacting partners in the protein G affinity pulldowns

Monolayer cells were harvested from three 150 mm plates of PPT1-CTAP-Puro or CTAP-Puro infected SH-SY5Y cells, grown to 80% confluency (1×10^8^ cells). Preparation of cytoplasmic extract from the cells was performed as previously described [8]. A volume of 200 μl packed IgG-Sepharose 6 Fast Flow resin (Amersham Biosciences) was used for batch purification of the cytoplasmic extract containing (TAP)-tagged protein complexes [8] with IgG binding domain. Lysates were incubated with resin for 16 h at 4 °C, washed thrice with 500 μl of 1× TBS-MNGZ (50 mM Tris–HCl pH 7.4, 150 mM NaCl, 2.5 mM MgCl_2_, 0.1% NP-40, 10% glycerol). Equal amounts of purified Protein-G affinity fractions (~20 µg each), from both empty CTAP-Puro and CTAP-PPT1-Puro were loaded onto gradient Bis-Tris 4–12% NuPAGE^®^ gels in MES SDS running buffer (Novex; Life Technologies, USA) and incubated with respective primary antibodies (anti-ATP5B, anti-DBH, anti-VCP and anti-β-actin; Section 1.4). The load control was assessed by probing the lysates (10% of the load) with the same primary antibodies.

## 2 Mass spectrometry analysis

### 2.1 Nano-LC/ESI/MS/MS analysis, data processing and SAINT analysis

Peptide analysis was performed on a *Q* Exactive Hybrid Quadrupole-Orbitrap mass spectrometer in positive ion mode for 182 min, with a selected mass range of 300–2000 mass/charge (*m/z*). For the survey scan, resolving power was set to 70,000 at *m*/*z* 200, maximum ion injection time 120 ms, dynamic exclusion of the selected precursor ions was set to 40 s, and the automatic gain control target value was 1.0e6. MS/MS data were acquired using the top 12 most abundant precursor ions with charge ≥2, as determined by the survey scan. These were selected with an isolation window of 1.2 *m/z* and fragmented via higher energy collisional dissociation with normalized collision energies of 27%. For the MS/MS scans, resolving power was set to 17.500 at *m*/*z* 200, maximum ion injection time 60 ms. MS/MS peak lists or spectral data were searched with the Mascot Daemon interface (version 2.2.0; Matrix Science) against the Swiss-Prot 55.1 database, with taxonomy set to *Homo sapiens*. Carbamidomethyl-Cys and Met oxidation were used as fixed and variable modifications, respectively. Mass tolerance of the precursor ions was set to ±ass to and of fragment ions to ±0.1 Da. The peptide charge was set to 1+, 2+, or 3+, and two missed tryptic cleavage sites were allowed. Only peptides which were ranked 1 and scored above the identity threshold score set at 99% confidence were withheld. SAINT analysis was performed as previously described [9,10], except that three biological replicates were used for the bait (PPT1) and negative control (empty CTAP-Puro vector). Proteins with an AvgPates were used for the bait (PPT1) and negative contrafter exclusion of contaminants (proteins that bind non-specifically to empty pES-CTAP-Puro vector alone, statistical contaminants (〈www.crapome.org〉) [11] and previously observed TAP contaminants) [12,13].

## 3 Bioinformatic and network analyses

### 3.1 Bioinformatic analyses and literature mining

Functional annotation of PPT1 IP for Gene Ontology_biological process (GO_BP) was performed by ClueGO, using the human genome as a background [14]. The following parameter settings were utilised: kappa ≥0.40, 3% threshold, GO levels 7–11 and a minimum of 2 genes per term (Fig. 4 in [1] and Supplementary Table 3).

Functional associations of PPT1 IP were analysed using FunCoup database v.3.0 (〈http://FunCoup.sbc.su.se〉). Based on 24 input genes (including PPT1 and PPT1 IP), 65 functionally coupled pairs with a very high confidence score (>0.8, scale 0−1) were identified (Fig. 4 and Supplementary Table 5).

### 3.2 Network analyses

**Fig. 3:**
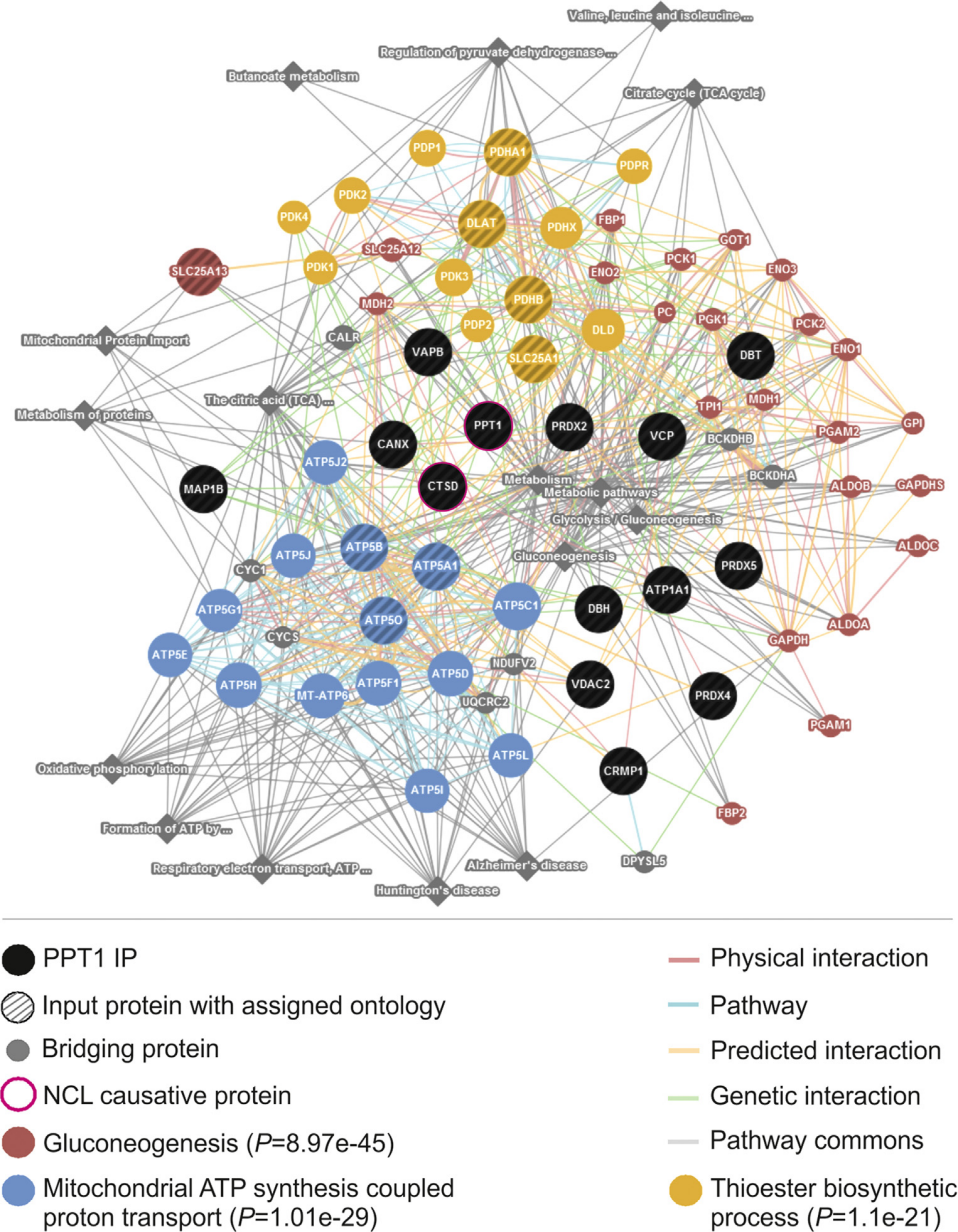
PPT1 interaction network. PPT1 interaction partners were linked to PPT1 using known physical and genetic interactions, pathway data and utilising knowledge of predicted interactions. The network was filtered according to GO BP criteria using *GeneMANIA* algorithm (www.genemania.org). Overall, 74 nodes of the network could be connected using 88 physical and 69 genetic interactions, 124 pathway and 133 predicted links. Pathway information from Pathway commons (Consolidated-Pathways-2013) was used to facilitate additional 16 links with varying weights (0.15–16.83%) to the PPT1 network. Nodes enriched with selected GO BP terms are indicated. Detailed information about the parameters of the network is presented in Supplementary Tables 4A–D.

**Fig. 4:**
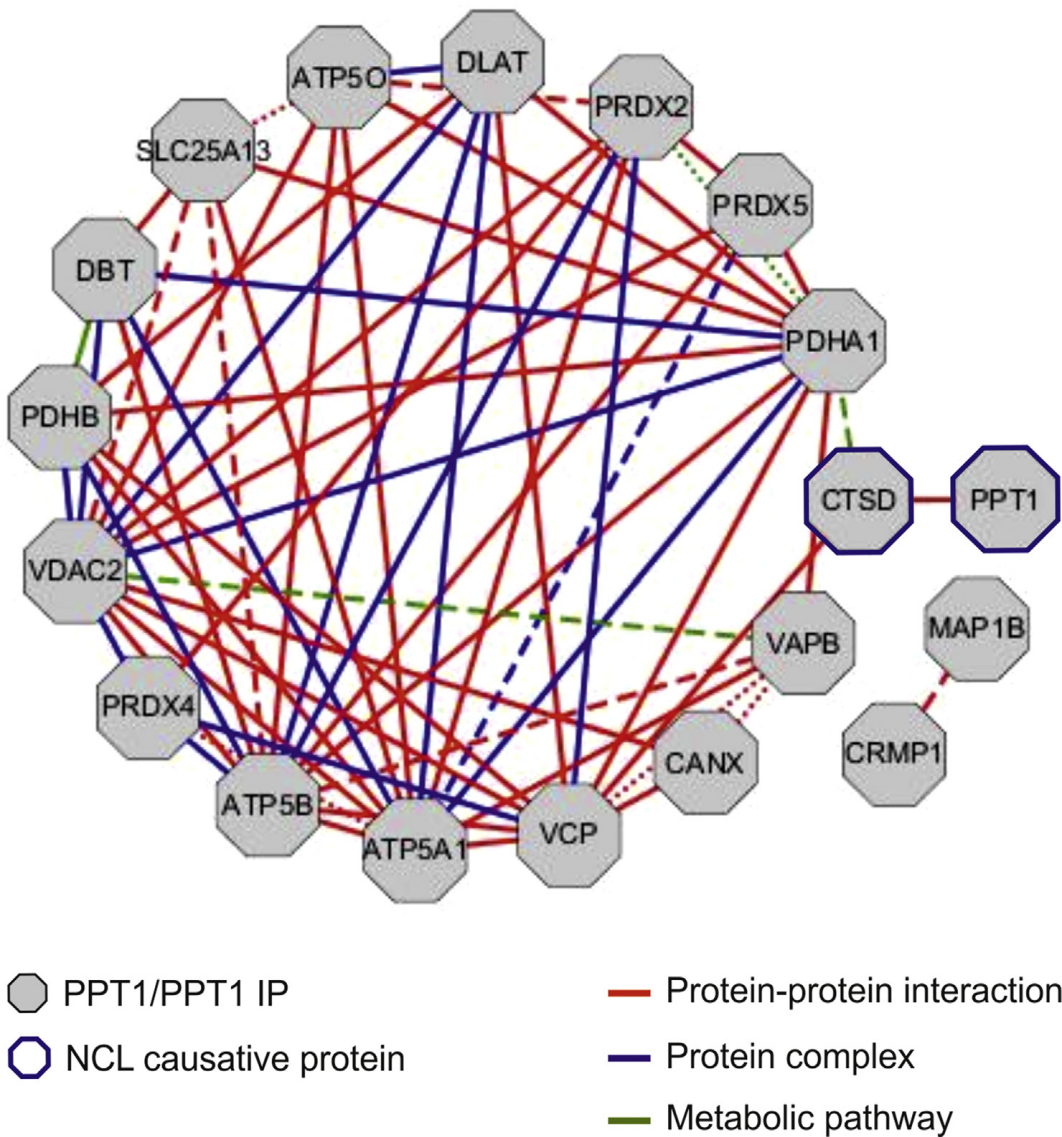
Global network analysis of PPT1 IP. Functional coupling of PPT1 IP was analysed by FunCoup database v.3.0 (http://FunCoup.sbc.su.se/). Based on 24 input genes (including PPT1 and PPT1 IP), 65 functionally coupled pairs with a very high confidence score (>0.8, scale 0−1) were identified. Dots, dashed and solid lines; indicate scores between (0.82–0.89), (0.9–0.95) and (>0.95), respectively. Details are provided in Supplementary Table 5, Supporting Information.

**Fig. 5:**
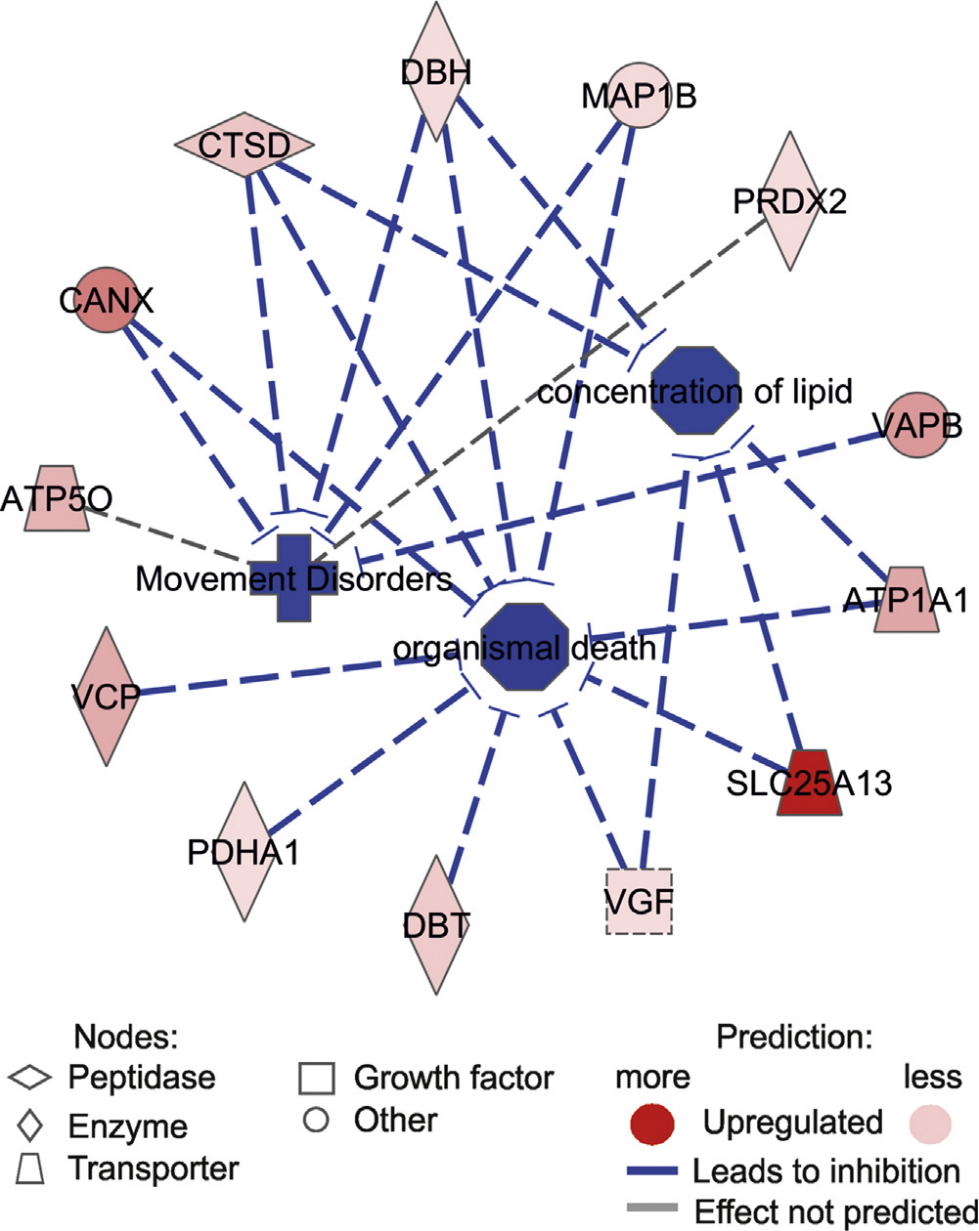
PPT1 IP association terms network. PPT1 interacting partners served as inputs into Ingenuity Pathways analyses (https://analysis.ingenuity.com/). Three terms: *inhibition of organismal death* (*Z*-score −3.109), *Movement disorders* (*Z*-score −2.209) and *concentration of lipid* (*Z*-score −2.133) were predicted to be altered in the PPT1 network.

The PPT1 and associated interacting partners were connected using Cytoscape software (〈http://www.cytoscape.org/〉) (Fig. 5 in Ref [1]) and Gene*Mania* (www.genemania.org (Fig. 3 and Supplementary Tables 4A–4D) [15,16]. The *latter indexes 2,104 associati*on networks containing 535,774,338 interactions mapped to 161,629 genes from 8 organisms, last update 10.07.2014) [17]. The network was constructed with physical, genetic, predicted and pathway interaction datasets listed by Gene*Mania*. For weighting of the networks we used GO BP algorithm from Gene*Mania*. Prediction of functional relationships in the PPT1 network was further performed using Ingenuity Pathway analysis (https://analysis.ingenuity.com/). The fold changes derived from spectral counts of SAINT analysis (all enriched for the PPT1 complex) were utilised as attributes in the analysis (Fig. 5 and Supplementary Table 5).

## Figures and Tables

**Fig. 2:**
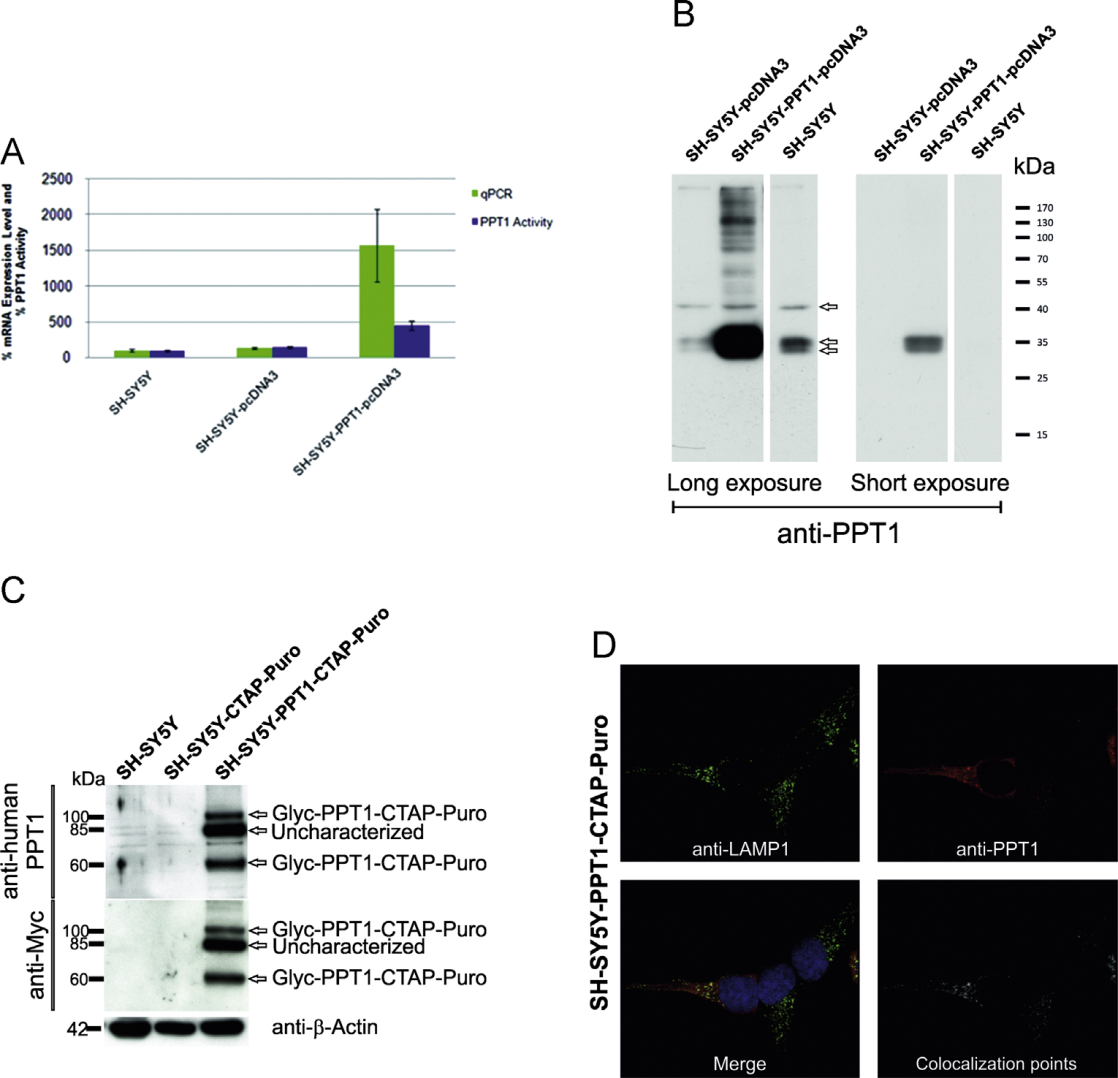
Expression of untagged human PPT1 and PPT1-CTAP-Puro in the stably transfected SH-SY5Y cells. (A) mRNA expression levels and enzyme activities of wild type human PPT1(CLN1) were assessed in SH-SY5Y, SH-SY5Y-pcDNA3 and SH-SY5Y-PPT1-pcDNA3 cells, respectively. SH-SY5Y cells stably expressing the untagged human PPT1 had a 10 and 4 fold difference in mRNA expression and enzyme activity, respectively, in comparison to the cells expressing the empty vector. (B) Western blotting analysis of the SH-SY5Y, SH-SY5Y-pcDNA3 and SH-SY5Y-PPT1-pcDNA3 cells using rabbit polyclonal anti-PPT1 (HPA021546, Sigma), indicated three faint bands of ~32, 35 and ~42 kDa detectable after long exposure from the lysates of not transfected cells only and cells expressing empty vector (arrows). Stronger band(s) running at ~32–39 kDa and one at ~42 kDa were detected in the human PPT1 expressing stable cells, after longer exposure. Only two weaker bands were detected from the lysates of the PPT1 expressing cells, and no signal was visible in lanes with SH-SY5Y and SH-SY5Y-pcDNA3 cells after short exposure. (C) Western blot analysis in empty SH-SY5Y, SH-SY5Y-CTAP-Puro and SH-SY5Y-PPT1-CTAP-Puro stable cells with anti- human PPT1 and anti-Myc antibodies revealed 3 bands at approx. 60, 80 and 100 kDa. Various glycosylated PPT1 forms have been observed in mouse fibroblasts and primary neurons [18]. Endogenous PPT1 protein is not detectable with rabbit polyclonal anti-GST-PPT1 [6]. Glyc is used as an abbreviation for glycosylated form of PPT1 (see Scifo et al. [1]). Uncharacterized- uncharacterized form of PPT1. Equal protein load was assessed with anti-β actin. (D) Immunodetection with rabbit polyclonal anti-PPT1 (1:500, red) and mouse monoclonal anti-LAMP1 [H4A3] (1:100, green) in SH-SY5Y-PPT1-CTAP-Puro cells. Nuclei were counter-stained with Hoechst 33342. PPT1 partially co-localises with the lysosomal marker, LAMP1 with a 47.9% overlap of the co-localisation signal. Images were acquired using a Leica SP8 confocal microscope (Wetzlar, Germany) and deconvoluted using Huygens Professional (Scientific Volume imaging). Analysis of co-localisation was performed with coloc2 plugin in FIJI image analysis software [7].
